# Revisiting Masculine and Feminine Grammatical Gender in Spanish: Linguistic, Psycholinguistic, and Neurolinguistic Evidence

**DOI:** 10.3389/fpsyg.2019.00751

**Published:** 2019-04-05

**Authors:** Anne L. Beatty-Martínez, Paola E. Dussias

**Affiliations:** ^1^ Center for Language Science, The Pennsylvania State University, University Park, PA, United States; ^2^ Department of Spanish, Italian and Portuguese, The Pennsylvania State University, University Park, PA, United States

**Keywords:** grammatical gender, gender assignment, language processing, language variation, Spanish

## Abstract

Research on grammatical gender processing has generally assumed that grammatical gender can be treated as a uniform construct, resulting in a body of literature in which different gender classes are collapsed into single analysis. The present work reviews linguistic, psycholinguistic, and neurolinguistic research on grammatical gender from different methodologies and across different profiles of Spanish speakers. Specifically, we examine distributional asymmetries between masculine and feminine grammatical gender, the resulting biases in gender assignment, and the consequences of these assignment strategies on gender expectancy and processing. We discuss the implications of the findings for the design of future gender processing studies and, more broadly, for our understanding of the potential differences in the processing reflexes of grammatical gender classes within and across languages.

## Introduction

Linguistic factors have long been known to modulate word identification. Of relevance for the work presented here, studies examining grammatical gender provide evidence that information at one point in a sentence is used to anticipate other information downstream. Grammatical gender is a widespread feature in many of the world languages. Simply put, it refers to “classes of nouns reflected in the behavior of associated words” (Hockett, 1958, p. 231; see also Comrie, 1999). Linguists agree that a language is said to have a grammatical gender system if there is evidence for gender outside the nouns themselves. One such type of evidence is gender agreement (Corbett, 1991). Examples (1a) and (1b) from Spanish illustrate this:

(1) (a) La televisión es roj**a**
  The_FEM_ TV_FEM_ is red_FEM_
  “The TV is red”
  (b) El teleférico es roj**o**
    The_MASC_ ski lift_MASC_ is red_MASC_
    “The ski lift is red”

In (1a), the form of the determiner is “*la”* and of the adjective is “*roj**a”*** because “televisión” is a feminine noun. In other words, the determiner and the adjective agree in gender with the noun they accompany. In (1b), the determiner “*el”* and the adjective “*roj**o***” agree with “teleférico” (a masculine noun).

A robust finding across languages with different gender systems (e.g., for Croatian, Costa et al., 2003; for French, Dahan et al., 2000; for German, Schmidt, 1986; for Italian, Bates et al., 1996; see Friederici and Jacobsen, 1999, for a review of early studies) is that when the gender of an article or adjective is congruent with that of the following noun, recognition of the noun is enhanced relative to a neutral baseline; when it is incongruent, recognition is delayed. This *gender congruency* effect has been reported in visual tasks (e.g., Jescheniak, 1999; Cubelli et al., 2005) and auditory tasks (e.g., Faussart et al., 1999; Dahan et al., 2000) and for languages with two genders (e.g., Barber and Carreiras, 2005) and more than two genders (e.g., van Berkum, 1996; Jacobsen, 1999). For instance, in Serbo-Croatian, lexical decision is faster for nouns preceded by adjective primes that match the nouns in gender than for those with mismatched preceding adjectives (Gurjanov et al., 1985). In addition, Cole and Segui (1994) reported that lexical decision is faster in French when primes are closed-class words (e.g., articles) relative to open-class words (e.g., adjectives), suggesting that the gender congruency effect changes as a function of word type. Results from Jakubowicz and Faussart (1998) have, in addition, shown that in a spoken lexical decision task, French adjectives phonetically marked for gender that intervened between an article and a noun (e.g., the adjective petit_MASC_ [pәti] /petite_FEM_ [pәtit], as in “le/*la petit chien,” the_MASC_/*the_FEM_ little_MASC_ dog_MASC_) do not increase the magnitude of the gender congruency effect relative to an invariant adjective without gender marking (e.g., the adjective pauvre_MASC/FEM_ [povʀ], as in “le/*la pauvre chien,” the_MASC_/*the_FEM_ poor dog_MASC_). This is significant because it highlights the central role of articles in setting gender agreement features for the entire noun phrase (Jakubowicz and Faussart, 1998). For Spanish, the language under investigation in this review, Lew-Williams and Fernald (2007) showed that Spanish-speaking children and adults exploit gender information on articles to facilitate the processing of upcoming nouns. Using the *looking-while-listening* procedure, Lew-Williams and Fernald presented participants with two-picture visual scenes, in which objects either matched or differed in grammatical gender. Target items were embedded in fixed carrier phrases (e.g., “encuentra *el*/*la*,” find the_MASC_/the_FEM_), and participants were instructed to find the named object. Results revealed that on different-gender trials, participants oriented their eyes toward target objects more quickly than on same-gender trials, yielding an anticipatory effect.

Importantly, studies reporting effects of prenominal gender marking on subsequent word identification have generally assumed that different gender classes (e.g., feminine and masculine in Spanish) modulate these effects with equal strength. Thus, with few exceptions (e.g., Gurjanov et al., 1985; Grosjean et al., 1994), studies have collapsed gender classes into a single analysis. Despite this general practice, in the work presented here, we discuss evidence from linguistic, psycholinguistic, and neurolinguistic studies, suggesting that grammatical gender classes may differentially contribute to the identification of nouns. Central to this proposal is the assumption that individuals of all language backgrounds are equipped with the ability to develop sensitivity to distributional information in language (Clayards et al., 2008; Gennari and MacDonald, 2009; Beatty-Martínez and Dussias, 2018). Our starting point is that words form relations along phonetic dimensions which contribute toward the creation of exemplar clusters. Categories are formed by placing exemplars in a conceptual space either closer to or further from each other depending upon the degree of dissimilarity of the members of a class (i.e., schematicity; Clausner and Croft, 1997). In the following sections, we provide evidence for this claim by examining distributional asymmetries between masculine and feminine gender in Spanish.

## On the Differential behavior of Masculine and Feminine Gender in Spanish

### Evidence From Monolingual Speakers

In Spanish, masculine has an unmarked or default status that sharply distinguishes it from feminine. One piece of evidence comes from loanwords, which are overwhelmingly assigned masculine gender. In a study by De la Cruz Cabanillas et al. (2007), 82% of the gendered loanwords in their corpus were masculine. In addition, masculine gender is also used in Spanish to refer to groups of individuals that include at least one male. As such, the noun phrase “los padres de Ana” (the_MASC_ fathers of Ana) can refer to Ana’s father and mother; “mis hijos” (my sons) can include daughters but not vice-versa; and “los estudiantes” (the_MASC_ students) can refer to groups of students in which all but one person are male.^1^ The unmarked status of Spanish masculine gender is further highlighted by agreement phenomena. When prepositions, conjunctions, and other non-gender marked words are used as nouns, they take masculine prenominals (e.g., reemplaza *este* “aunque” por *un* “sin embargo”, replace this_MASC_ “still” for a_MASC_ “nevertheless”) and masculine determiners are used in nominalizations (e.g., “*el* fumar mata,” the_MASC_ smoking kills). A study by Eddington and Hualde (2008) presented intriguing evidence showing that native speakers of Spanish make errors when assigning gender to certain Spanish feminine nouns. In Spanish, the phonological pattern most typically associated with feminine gender is the presence of a final /a/ phoneme, illustrated in nouns such as “*casa”* (house), “*mesa”* (table), “*arpa”* (harp), and “*águila”* (eagle). Endings for masculine nouns include the vowels *-o* and *-e*, as well as a number of consonants (e.g., *-l* [“*caracol,*” snail_MASC_], *-n* [“*tren*,” train_MASC_], *-j* [“*reloj*,” watch_MASC_]), reflecting the fact that Spanish masculine phonological endings are less restricted. Feminine nouns, however, have an additional complicating rule. When the onset of a Spanish feminine noun is a stressed /a/, singular definite determiners (“*la*,” the_FEM_) and determiners ending in /-una/ (“*una*,” a_FEM_; “*alguna*,” some_FEM_; “*ninguna*,” none_FEM_) must carry masculine gender if they immediately precede the noun.^2^ The reason appears to be a phonetic infelicity involving word-final /a/ immediately followed by stressed word-initial /a/. This is shown in the examples (2a) and (2b) below:

(2) (a) *un**a*** costos**a** arp**a**
  a_FEM_ expensive_FEM_ harp_FEM_
  “an expensive harp”
  (b) *un* arp**a** costos**a**
    a_MASC_ harp_fem_ expensive_fem_
    “an expensive harp”

What Eddington and Hualde (2008) found is that this variation produces confusion in native speakers, which results in the (incorrect) use of masculine prenominal modifiers appearing to the left of these nouns and feminine post-nominal modifiers appearing to the right:

(3) (a) Echa *tod**o** el* agu**a** frí**a** en el barreño
  pour all_MASC_ the_MASC_ water_FEM_ cold_FEM_ in the basin
  “pour all the cold water in the basin”
  instead of
  (b) Echa *tod**a** el* agu***a*** frí***a*** en el barreñopour all_FEM_ the_MASC_ water_FEM_ cold_FEM_ in the basin
    “pour all the cold water in the basin”
    (Eddington and Hualde, 2008, p. 4)

Psycholinguistic evidence also highlights the unmarked status of Spanish masculine gender. Domínguez et al. (1999) found that for masculine and feminine words closely matched in frequency, mean reaction times during a lexical decision task were shorter for the masculine than the feminine forms. Another source of linguistic evidence comes from studies on Spanish gender acquisition. Pérez-Pereira (1991) observed that monolingual Spanish-speaking children made use of a noun’s phonological shape (i.e., whether nouns ended in -*a* or -*o*) when assigning gender to determiners. However, Pérez-Pereira also observed that children were more likely to assign masculine gender to nouns with irregular (i.e., ambiguous) phonological cues, suggesting a masculine default strategy in gender assignment (Harris, 1991). One question raised by these results is whether the preference for masculine gender stems from distributional frequency differences in language input to children. Smith et al. (2003) examined a corpus of child-directed speech and developed a connectionist model of gender assignment to mirror the type frequency patterns to which a child is exposed over time. Analysis of the corpus revealed an equal number of masculine and feminine nouns. However, upon closer inspection, distributional frequency differences between regular (i.e., nouns ending in -*a* or -*o*) and irregular nouns emerged: “while regular feminine nouns were slightly more frequent than regular masculine nouns, irregular masculine nouns outnumbered irregular feminine nouns by roughly 2 to 1” (Smith et al., 2003, p. 306). The model, which was incrementally trained on this input, produced a similar bias toward masculine gender when tested on novel words, suggesting that the frequency distribution, particularly the interaction between gender and word form ambiguity, plays a direct role in gender assignment.

A potential limitation of the Smith et al. (2003) study is that it did not examine the role of phonological factors beyond the word-final phoneme in determining gender assignment. Contrary to previous claims in the literature (Harris, 1985; Roca, 1989), the correspondence between the gender of a noun and its phonological shape is not fortuitous. Eddington (2002) used an exemplar-based model to determine the gender of a noun based on its phonological shape. The database for the simulation included a list of highly frequent nouns in Spanish taken from Juilland and Chang-Rodríguez’s (1964) frequency estimates. Each noun was encoded to include its phonemic makeup (e.g., the word’s final phoneme) and the syllabic structure of the penultimate and final syllables. When the penultimate rhyme and final syllable variables were included in the model, the algorithm successfully assigned gender to 95% of nouns. To determine whether native speakers were able to exploit the same systematic correspondences as the model, Eddington tested a group of monolingual Spanish-speaking adults on a gender assignment task using novel words with ambiguous endings (i.e., final phonemes other than -*a* and -*o*). The results produced a clear bias toward masculine gender assignment, replicating previous findings. Notably, an assessment of success and error rates for each of the variables confirmed a high degree of association between the model and native speakers’ intuitions.

Altogether, the Eddington (2002) results suggest that speakers establish and make use of phonological factors besides word-final phonemes to assign grammatical gender. Eddington suggests that the structure of the nouns themselves provides an explanation for speakers’ bias toward masculine due to a markedness asymmetry between the two genders. In a marked/unmarked relation, the marked member of the opposition (i.e., feminine gender) has a densely clustered category, settling on a tighter range of variance. The unmarked category (i.e., masculine gender), on the other hand, covers a wider range of configurations (Greenberg, 1966). “[W]hat this means for gender is that a random throw of the dart onto a map of nouns organized according to phonological similarities, has a much higher probability of landing in a neighborhood of masculine nouns, even if they do not dominate feminine nouns numerically” (Eddington, 2002, p. 66). We return to the role of morphological markedness on gender processing in the section devoted to electrophysiological evidence.

### Evidence From Bilingual Speakers

The evidence presented above raises the question of whether Spanish masculine and feminine articles differentially affect the time course of noun processing. One potential disadvantage of the current monolingual work is that most studies have employed offline grammaticality judgments or speech elicitation experiments with novel words out of context, which are artificial tasks. In this respect, bilingualism can be used as a tool to examine questions that are sometimes not easily studied with monolingual populations. We adopt a broad definition of bilingualism to include speakers who actively use two or more languages, regardless of whether those languages were acquired in early childhood or later in life. In this section, we will review gender assignment strategies in bilingual speakers with a special emphasis on codeswitching^3^, the alternation between languages within and between utterances in bilingual discourse. Like monolinguals, bilingual speakers of Spanish and another language have been shown to have a similar preference to assign masculine gender to determiners for loanwords (Smead, 2000; Aaron, 2015), with the exception of established loanwords that are strongly morphologically integrated in Spanish (e.g., “la troca,” the truck; Clegg and Waltermire, 2009). However, a characteristic of many bilingual communities of the Spanish-speaking world is to routinely switch between Spanish and another language when speaking to other bilinguals. We propose that codeswitching provides a special testbed for the study of distributional asymmetries in gender assignment while circumventing some of the obstacles outlined above (Myers-Scotton and Jake, 2015). Specifically, codeswitched noun phrases (NPs) are abundant in Spanish-English codeswitched speech (Timm, 1975; Pfaff, 1979; Poplack, 1980). Because “mixed” NPs (i.e., NPs that appear in two languages) are highly frequent in the everyday speech of some bilingual populations, they provide a valuable alternative for examining gender assignment strategies as a means to reveal the underlying mechanisms that are responsible for asymmetrical distributions. How so? Because when bilinguals codeswitch, they make opportunistic decisions about how to integrate the two linguistic systems on the fly (Green and Wei, 2014). Their production choices provide, in turn, a window on speakers’ prior linguistic experience (Beatty-Martínez et al., 2018a). For example, corpus studies on Spanish-English codeswitching have noted that bilinguals are more likely to produce mixed NPs with Spanish determiners and English nouns (e.g., “*el* dog,” the_SPAN_ dog_ENG_) over mixed NPs with the opposite configuration (e.g., “the *perro*,” the_ENG_ dog_SPAN_; Jake et al., 2002; Valdés Kroff, 2016; Beatty-Martínez et al., 2018a; Królikowska et al., 2019; cf. Blokzijl et al., 2017). Similarly, many studies have reported a masculine tendency in the assignment of grammatical gender for Spanish-English mixed NPs similar to the sentences in (4a) and (4b) below (Montes-Alcalá and Lapidus Shin, 2011; Valdés Kroff, 2016; cf. Liceras et al., 2008). What makes this observation particularly interesting is that many English nouns in mixed NPs have a clear Spanish translation equivalent, so the opportunity to examine how these switches are integrated in spontaneous conversation sheds light on the asymmetrical relationship between masculine and feminine by revealing which linguistic mechanisms are at play in a way that is otherwise obscured in monolingual speech.

(4) (a) La señora colocó *un* **knife** next to every plate
  The woman placed a_MASC_ knife_MASC_
  (b) La señora colocó *un*  **spoon** next to every plate
    The woman placed a_MASC_ spoon_FEM_

Current work in our research group is aimed at determining the extent to which codeswitching patterns are community-specific or generalizable across different speech communities of the Spanish-speaking world. To explore this issue, we have designed a conversational paradigm to obtain spontaneous speech samples of bilingual speakers (Beatty-Martínez and Dussias, 2017; Beatty-Martínez et al., 2018a). In the task, participants are assigned the role of director and are instructed to communicate to a matcher addressee how to arrange a series of images printed on a map. To maximize ecological validity, no language restrictions are imposed; that is, participants are free to use whichever language they choose. The project resulted in four comparable corpora of over 100 Spanish-English bilingual young adults from four linguistically distinct interactional contexts (San Juan (PR), El Paso (TX), State College (PA), and Granada (Spain)). Based on these data, Królikowska et al. (2019) asked whether all groups showed the attested preference for masculine determiners before switching to an English noun, regardless of the gender of the translation equivalent.

**Figure 1:**
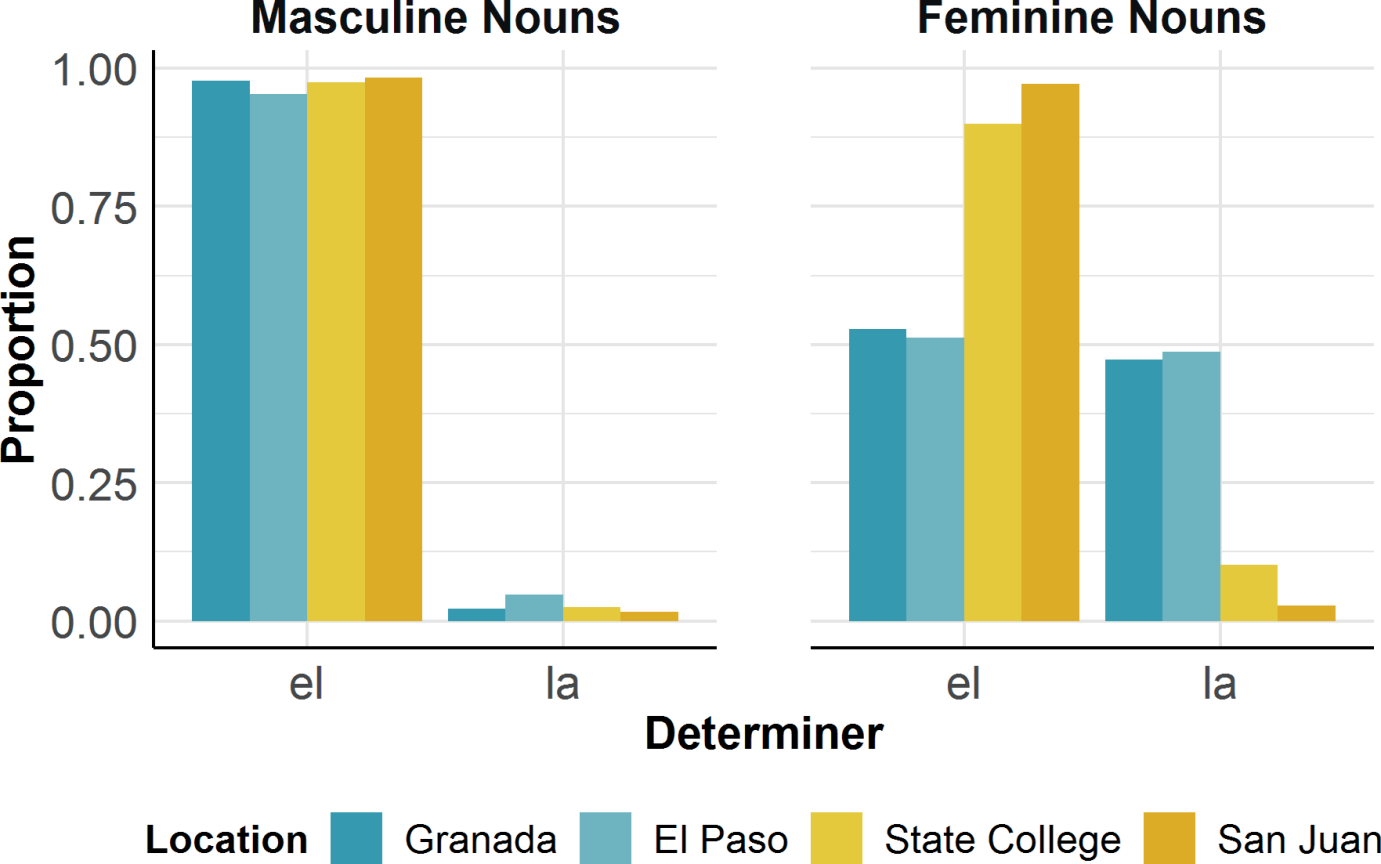
Distribution of mixed NPs across four bilingual communities in Królikowska et al. (2019).

Figure 1 illustrates an asymmetric relation between masculine and feminine grammatical gender assignment across all four groups. For bilinguals in San Juan and State College, the data show an overwhelming preference for masculine determiners, regardless of the grammatical gender of the Spanish translation equivalent. Moreover, while bilinguals in Granada and El Paso also exhibited higher rates of masculine determiners overall, they also produced higher rates of feminine determiners than the other two groups. Specifically, masculine and feminine determiners were produced at similar rates for nouns with feminine translation equivalents (e.g., “*la* spoon,” the_FEM_ spoon_FEM_).

**Figure 2:**
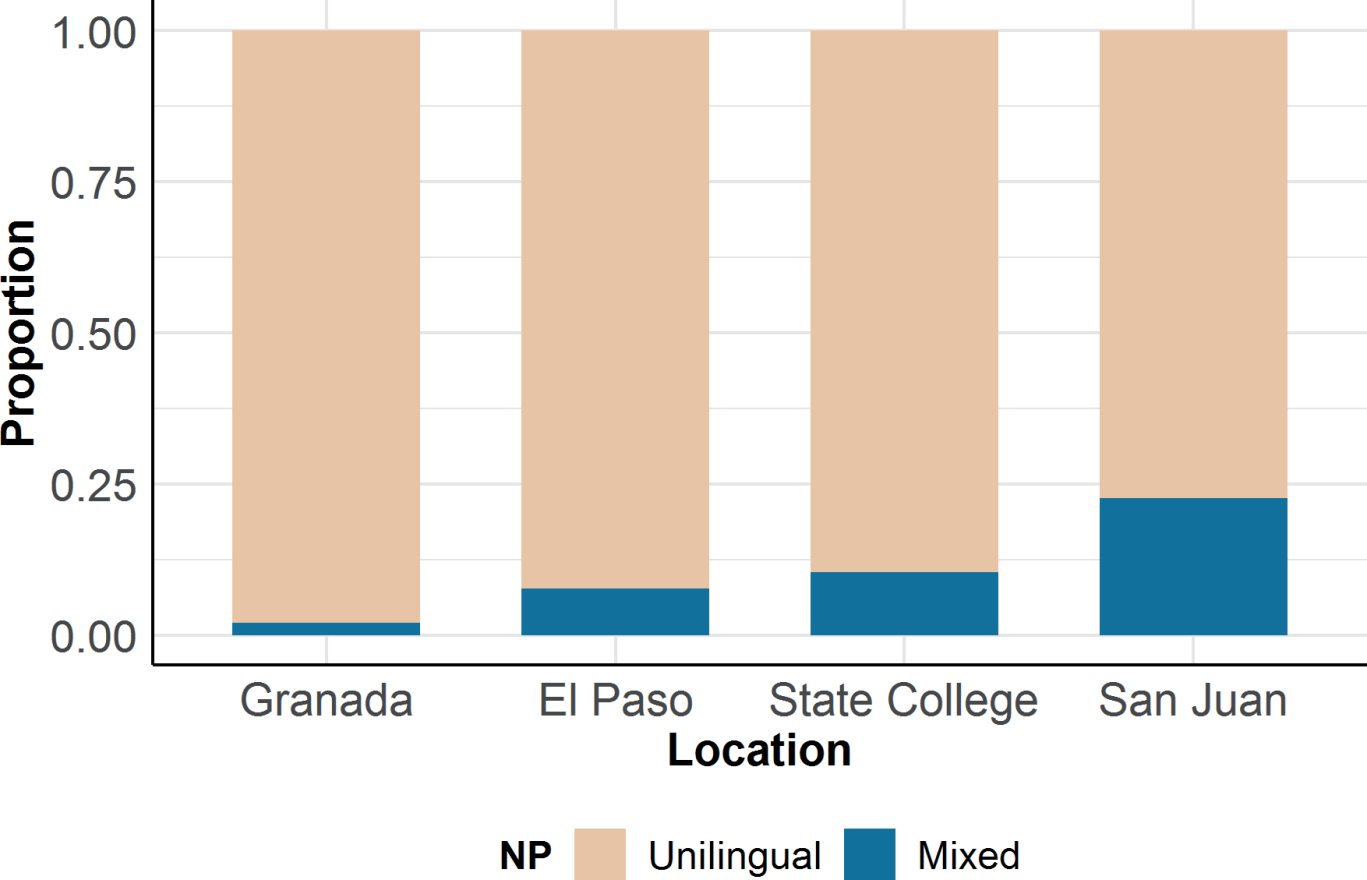
Rates of expression of unilingual and mixed NPs across four bilingual communities in Królikowska et al. (2019).

Although more work is needed to unpack these results, one possible explanation for the variability between these four contexts is that bilinguals from these communities exhibit different rates of codeswitching overall. Figure 2 depicts rates of unilingual (e.g., English: “the dog”; or Spanish: “el perro”) and mixed (e.g., “el dog”) NPs across the four testing locations. Bilinguals from San Juan had the highest rate of codeswitching at almost 24%, while bilinguals from Granada had the lowest at 2%. Therefore, one possibility is that the more the bilinguals engage in codeswitching, the greater the tendency to assign the default masculine gender to mixed NPs. This is an important observation that supports previous claims that codeswitching preferences reflect community norms and are therefore not necessarily generalizable across bilingual populations, even when examining the same language pair (Poplack, 1988; Aaron, 2015; Beatty-Martínez et al., 2018a).

Because most English words differ from typical Spanish words with respect to their phonological shape (Clegg, 2010; Butt and Benjamin, 2013), it is difficult to determine whether the masculine default strategy is, at least to some degree, driven by phonological factors (Poplack et al., 1982; DuBord, 2004; Montes-Alcalá and Lapidus Shin, 2011). Below, we consider two recent studies that examined how the phonological shape of nouns from different source languages (i.e., Basque and Purepecha) can influence the choices speakers make in terms of the choice of gender assignment.

Parafita Couto et al. (2015) examined grammatical gender assignment strategies of Spanish-Basque NPs in naturalistic speech and auditory judgement data. Basque differs from Spanish and English in its morphological behavior and NP word order. In Basque, the definite determiner -*a* appears suffixed to the noun (e.g. “sagarr-*a,”* the apple) which is coincidentally homophonous with the regular feminine endings in Spanish (e.g., “la manzana”). The naturalistic data indicated a preference for the feminine determiner when it was congruent with the Basque phonological ending -*a*, providing converging evidence for the role of a noun’s phonological shape in gender assignment.

In a similar study, Bellamy et al. (2018) examined gender assignment in Spanish-Purepecha mixed NPs using a production task and an online acceptability judgement task. Like Basque, Purepecha has bound suffixes terminating in -*a* that coincides with phonological cues to feminine gender assignment in Spanish. In the production task, participants overwhelmingly preferred to use masculine determiners, irrespective of the noun ending or Spanish translation equivalent. In the acceptability judgement task, participants also preferred masculine assignment except in cases where nouns ended in -*a*. Bellamy et al. interpreted this result to indicate that orthography can lead speakers to re-interpret the -*a* ending suffix, a marker of feminine gender. Furthermore, the discrepant findings of these tasks provide evidence that the modality of the task can influence gender agreement strategies in Spanish speakers. Taken together, these studies highlight how preferences in gender agreement are susceptible to both cross-language effects and the type of task. In the next section, we consider how bilingual language experience can lead to the same adaptive consequences in predictive processing.

## Implications for Language Processing

### Eye-Tracking Evidence

We discussed earlier how the study of codeswitching provides a unique lens through which the differential status of masculine and feminine gender in Spanish can be examined. The distributional patterns outlined in the “Evidence From Bilingual Speakers” section on the use of grammatical gender in Spanish-English mixed noun phrases raise the question of whether the asymmetries observed in Spanish-English mixed NPs has consequences for the comprehension system, as would be predicted by experience-based models of language processing (e.g., MacDonald, 2013; Dell and Chang, 2014). Initial results indicate that they do. In a series of eye-tracking experiments, Valdés Kroff et al. (2016) capitalized on competitor (Allopenna et al., 1998) and anticipatory (Lew-Williams and Fernald, 2007) effects reported in studies of spoken language processing using the visual world paradigm (Tanenhaus et al., 1995) to examine whether the overwhelming preference for the Spanish masculine article in codeswitched noun phrases had any consequences for the comprehension system. Target items in the codeswitching condition were made up of a Spanish preamble (“Encuentra *el*/*la*,” find the_MASC_/the_FEM_) followed by an English target noun, yielding mixed NPs such as “*Encuentra el candy*.” To provide a test of the hypothesis that speakers exploit feminine but not masculine cues on determiners to anticipate upcoming nouns, they incorporated an additional manipulation. The mixed NPs contained pairs of items that were phonological competitors in English. For example, *candy* and *candle* overlap phonologically in the first syllable [kæn], but critically their Spanish translations differ in grammatical gender; *candy* is English for dulce_MASC_ or caramelo_MASC_ and *candle* is English for vela_FEM_. Because in mixed NPs, the pattern from corpus studies suggests that the definite article *el* surfaces with English nouns whose Spanish translations are both masculine and feminine, the prediction was that the gender information encoded in the article would not facilitate the processing of sentences such as “Encuentra *el* candy.” Instead, the presence of phonological competitors should evince a competitor effect, and this is precisely what they found. When a masculine article was heard in the presence of the picture pair *candle*-*candy*, the results showed a clear competitor effect, suggesting that the masculine article *el* was not informative when bilinguals were asked to select a noun. In other words, it functioned as a default article in Spanish-English codeswitching. When a feminine article was heard in the presence of the same two pictures (i.e., “Encuentra *la* candle”), the results showed a different pattern. Participants failed to display an anticipatory effect and instead experienced an extended delay in processing for target items that did not match in grammatical gender (e.g., *la* candy) likely reflecting the rarity of this type of mixed NP in Spanish-English codeswitching.

### Electrophysiological Evidence

Thus far, we have argued that the distributional asymmetry between masculine and feminine gender reflects underlying differences in the representation of the two genders. In this section, we turn to electrophysiological studies of grammatical gender to examine possible differences in processing and representation for masculine and feminine nouns in unilingual and mixed NPs. In contrast to behavioral measures, which reflect the cumulative outcome of several processes, the event-related potentials (ERPs) technique can provide high temporal resolution indices at different stages of processing, which is reflected in modulations of distinguishable components. Importantly, ERPs have been found to be modulated by different linguistic processes, including morphological markedness (Deutsch and Bentin, 2001; Kaan, 2002; Alemán Bañón and Rothman, 2016), making this technique particularly suitable to uncover potential differences in the processing of masculine and feminine grammatical gender.

ERPs have been widely employed to investigate the time course of noun phrase grammatical gender processing in both monolingual (Wicha et al., 2004; Barber and Carreiras, 2005; Caffarra and Barber, 2015) and bilingual (Caffarra et al., 2017a) speakers. The general finding is that grammatical gender violations in Spanish elicit a biphasic pattern, consisting of a Left Anterior Negativity (LAN) around 300 ms after stimulus onset and a subsequent P600 after 500 ms.^4^ The LAN effect has been suggested to reflect initial processes for detection of a morphosyntactic violation (Osterhout, 1997). The P600 effect has been linked to processes of reanalysis and repair of syntactic anomalies (Osterhout and Holcomb, 1992; Friederici et al., 1996; Kaan et al., 2000).

Caffarra and Barber (2015) investigated whether distributional gender cues conveyed by Spanish noun endings (i.e., -*a* for feminine and -*o* for masculine) can influence gender processing in native Spanish speakers. Nouns with regular endings elicited a greater sustained negativity around 200 ms after the stimulus onset suggesting that Spanish speakers are sensitive to noun endings (see Halberstadt et al., 2018 for related findings with second language speakers of Spanish using eye-tracking methodology). Notwithstanding, a LAN-P600 biphasic pattern was similarly reported for gender violations for both regular and irregular nouns. Based on these findings, the authors concluded that grammatical gender agreement processes rely mostly on the representation of gender, regardless of distributional gender cues conveyed by noun endings. Using the same paradigm, Caffarra et al. (2017a) replicated these results with Spanish-Basque bilinguals but observed that participants who reported using Spanish more regularly were able to detect violations for irregular nouns earlier and more easily than those who were Basque dominant. These results highlight the role of regular correspondence between the word form and a specific gender class and, more broadly, of experience that users have with language in category learning and representation. At the same time, these findings also suggest that lexical representations may become more entrenched with greater language experience, resulting in more efficient processing.

A few studies have investigated gender agreement processes of masculine and feminine genders separately using ERPs. Alemán Bañón and Rothman (2016) examined the brain’s sensitivity to noun-adjective agreement violations during online sentence comprehension. ERPs were time-locked to adjectives appearing predicatively in relative clauses. In their design, half of the items were masculine and the other half were feminine. They found that both types of gender agreement violations yielded robust P600 effects albeit earlier for feminine-marked adjectives. Alemán Bañón and Rothman interpreted the difference in latency as evidence that violations realized on marked predicates are easier to detect and thus revised more quickly, consistent with previous work on syntactic processes of diagnosis and repair (e.g., Friederici, 1998; Kaan, 2002). Notwithstanding, the processing of noun-adjective agreement has been shown to differ from the processing of gender assignment with nouns (Dewaele and Véronique, 2001; Barber and Carreiras, 2003; Kupisch et al., 2013), and while adjectives and nouns have overlapping cues to gender, there are differences in marking consistency between the two elements. It follows that a manipulation of gender agreement ultimately addresses a different question than the one we ask here: If the attested distributional asymmetries in gender assignment reflect differences intrinsic to the structure of nouns (e.g., Eddington, 2002) and speakers have been shown to attend to and make use of these cues in production, what consequences do these adjustments have for lexical processing and representation?

**Figure 3:**
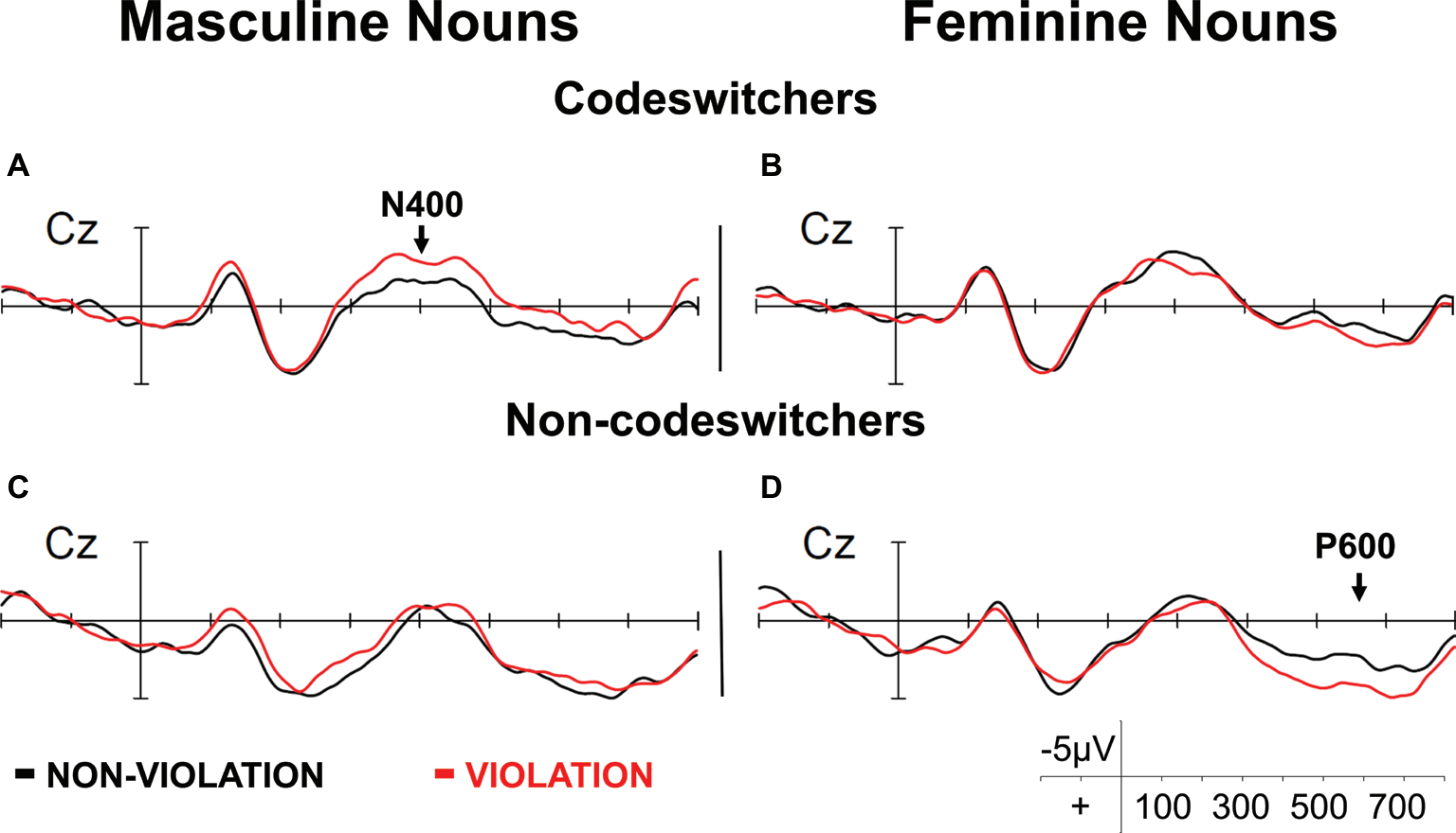
ERPs time-locked to the onset of masculine **(A,C)** and feminine **(B,D)** nouns for codeswitchers **(A,B)** and non-codeswitchers **(C,D)** at the electrode site Cz. Figure adapted from Beatty-Martínez and Dussias, 2017, Copyright (2017), with permission from Elsevier.

To our knowledge, only two studies have compared gender processes in nouns as a function of their gender in Spanish. Beatty-Martínez and Dussias (2017) examined gender processing in mixed NPs for bilinguals differing in codeswitching experience (i.e., codeswitchers and non-codeswitchers). In their design, the gender of the target noun (i.e., the gender of its translation equivalent in Spanish; e.g., masculine: “knife,” cuchillo_MASC_ or feminine: “spoon,” cuchara_FEM_) was manipulated such that it either agreed in gender with the preceding determiner (congruent condition: “*el* knife,” the_MASC_ knife_MASC_) or not (incongruent condition: “*la* knife,” the_FEM_ knife_MASC_). For codeswitchers, masculine targets in incongruent mixed NPs (e.g., “*la* knife”) were more difficult to integrate relative to masculine targets in congruent mixed NPs (e.g., “*el* knife”; Figure 3A). Importantly, incongruent mixed NPs with masculine determiners (e.g., “*el* spoon”) did not result in processing difficulties (Figure 3B). The authors interpreted this result as evidence for bilinguals’ sensitivity to distributional codeswitching patterns (i.e., incongruent mixed NPs with feminine determiners are rarely attested in naturalistic codeswitching; Valdés Kroff, 2016; Beatty-Martínez et al., 2018a). Non-codeswitchers, on the other hand, only showed sensitivity to agreement violations for mixed NPs involving feminine translation equivalents: incongruent mixed NPs (e.g., “*el* spoon”) elicited a P600 effect (Figure 3D). While the absence of the P600 in incongruent mixed NPs involving masculine translation equivalents (e.g., “*la* knife”; Figure 3C) is likely due to substantial variability in participants’ responses (McLaughlin et al., 2010; Qi et al., 2017), these differences in themselves are likely indications of the differential representation of masculine and feminine gender.

**Figure 4:**
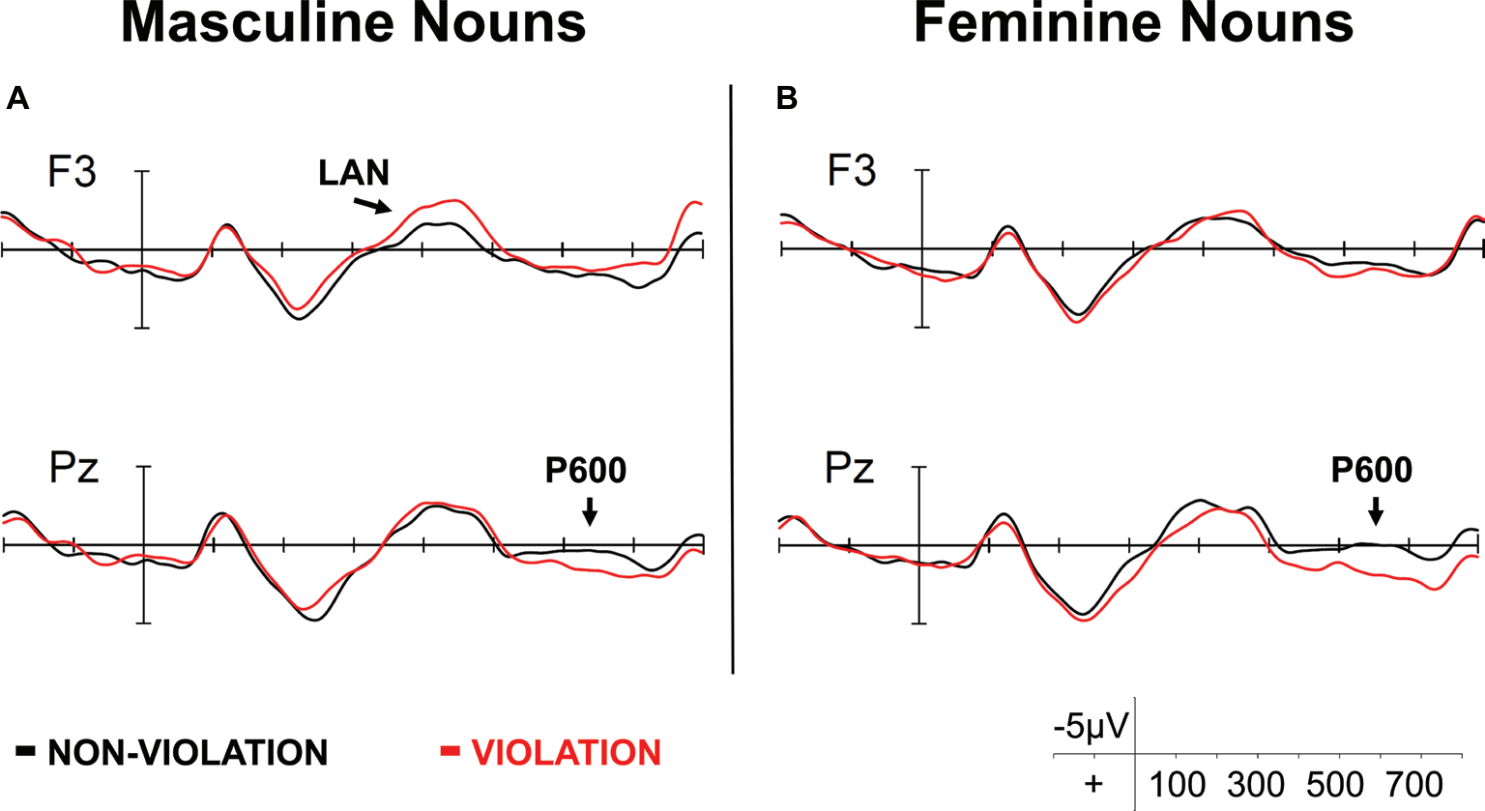
ERPs time-locked to the onset of masculine **(A)** and feminine **(B)** nouns at F3 and Pz electrode sites adapted from Beatty-Martínez et al. (2018b).

An alternative explanation proposed in the Caffarra et al. (2017a) study is that knowledge and usage of a second language may influence the strength of gender lexical representation, and that therefore, bilinguals may not rely on gender features in the same way as native speakers. We would like to take this proposal a step further and assume that variability in grammatical gender processing exists even among monolinguals processing their native language (see Tanner et al., 2014, for a discussion on “native-like” processing). We consider a recent study whose findings may provide insights into this issue. Beatty-Martínez et al. (2018b) examined the electrophysiological correlates of masculine and feminine gender violations in native monolingual Spanish speakers. Specifically, ERPs were recorded while participants read sentences in Spanish that were either well-formed or contained grammatical gender violations. Half of the target nouns were masculine (e.g., “cuchillo,” knife) and half were feminine (e.g., “cuchara,” spoon) in gender. When collapsed across gender, the gender violation showed the classical LAN-P600 biphasic pattern. However, splitting the data by noun gender revealed different ERP patterns to masculine and feminine gender. Responses to masculine grammatical gender violations had far greater variability and showed a reduced P600 (Figure 4A). This is consistent with previous studies showing reduced sensitivity to morphological violations involving unmarked elements (Deutsch and Bentin, 2001; Kaan, 2002; Alemán Bañón and Rothman, 2016). As illustrated in Figure 4B, feminine gender violations elicited a more robust P600 response that was modulated by vocabulary knowledge: individuals with higher Spanish vocabulary were more sensitive to grammatical gender violations with feminine nouns. We interpret this finding to suggest that as vocabulary increases, so does the strength of the representation of noun clusters, supporting the more general idea that experience with language affects the structure of categories and has an impact on cognitive representations (e.g., Bybee, 2010). Together, the results in this section provide support for a differential representation between masculine and feminine gender by demonstrating that variability in gender processing exists even among groups traditionally assumed to be homogenous.

## Conclusion

The main objective of this paper was to examine distributional asymmetries between masculine and feminine gender, the resulting biases in gender assignment, and the consequences of these assignment strategies on gender expectancy and processing. While the available evidence is not conclusive, a striking feature that emerges from this review is an underlying difference in the representation and processing of masculine and feminine gender in Spanish. What does this difference mean for our understanding of grammatical gender? The processing results reported here, together with the acquisition data, suggest that assumptions made in past processing literature, which have treated different gender classes similarly, is unwarranted. Grammatical gender has been extensively studied in a wide variety of disciplines, yet there is often little crosstalk between different fields of study. Within the second language processing literature for example, grammatical gender has served as the benchmark of native-like attainment, with some studies reporting differential sensitivity in the second language and others arguing against such differences. The evidence presented here contributes to this debate through a consideration of distributional factors in explaining differences in grammatical gender processing.

While distributional asymmetries are not necessarily language specific, we caution against generalizing the specific biases arising in Spanish across other gendered languages for several reasons. First, languages differ with respect to how gender classes are distributed. While masculine and feminine gender are distributed approximately equally in Spanish (Bull, 1965), other languages with a binary gender system have a less balanced distribution (e.g., about 3:1 ratio for masculine and neuter nouns in Dutch; van Berkum, 1996). Gendered languages also differ in the degree to which gender assignment can be made in terms of phonological shape or morphological composition. For example, historical sound change in French turned regular feminine endings to schwas (e.g., “fenestra → fenêtre,” window), resulting in greater phonic ambiguity in the endings of masculine and feminine nouns (Nelson, 2005). Moving forward, we suggest that more interdisciplinary studies are needed to exploit the consequences of distributional regularities on language processing. More broadly, processing research must proceed from a distinct set of assumptions regarding the status of grammatical gender, adopting an approach in which gender is not viewed as a single concept but rather recognized as a complex and granular phenomenon, whose processing reflexes may exhibit surprising asymmetries.

## Author Contributions

Both authors have contributed equally to the manuscript and approved it for publication.

### Conflict of Interest Statement

The authors declare that the research was conducted in the absence of any commercial or financial relationships that could be construed as a potential conflict of interest.

## Footnotes

^1^Although not the focus of the current view, there is also some evidence of grammatical gender asymmetries when referring to human beings (or animate beings in general). More specifically, some have argued that the generic use of masculine forms in gendered languages may lead to biased representations of gender during language processing (Spanish: Carreiras et al., 1996; see Gabriel and Gygax, 2016, for a detailed discussion on this issue).

^2^While exceptions exist (e.g., ‘*el* dí*a*’, the_MASC_ day_MASC_; ‘l*a* man*o*’, the_FEM_ hand_FEM_), −*a* and -*o* endings been shown to be highly correlated with masculine and feminine gender respectively (Bull, 1965; Harris, 1991; Eddington, 2002; Clegg, 2010). We refer to -*a* and -*o* endings as predictor variables for gender assignment rather than gender morphemes. These variables are probabilistic, some being more reliable than others (see Table 1 in Eddington, 2002, for a list of other phonemic variables interpreted as relevant for gender assignment).

^3^For ease of exposition, we adopt a broad definition of codeswitching to include single word and multiword constituents.

^4^We note that the LAN is not consistently observed across studies examining morphosyntactic violations (e.g., Wicha et al., 2004; Alemán Bañón et al., 2012). Recent reports have questioned whether this effect even exists (Tanner, 2015), although others disagree (Molinaro et al., 2015; Caffarra et al., 2017b).
